# Enterprise Microblogging to Augment the Subinternship Clinical Learning Experience: A Proof-of-Concept Quality Improvement Study

**DOI:** 10.2196/mededu.9810

**Published:** 2018-08-21

**Authors:** Irsk Anderson, Oliver Hulland, Jeanne M Farnan, Wei Wei Lee, Debra Milton, Vineet M Arora

**Affiliations:** ^1^ Department of General Internal Medicine Pritzker School of Medicine University of Chicago Chicago, IL United States; ^2^ Department of Emergency Medicine Yale School of Medicine Yale University New Haven, CT United States

**Keywords:** social media, medical education, microblogging platform, distance learning

## Abstract

**Background:**

Although the Clerkship Directors in Internal Medicine (CDIM) has created a core subinternship curriculum, the traditional experiential subinternship may not expose students to all topics. Furthermore, academic institutions often use multiple clinical training sites for the student clerkship experience.

**Objective:**

The objective of this study was to sustain a Web-based learning community across geographically disparate sites via enterprise microblogging to increase subintern exposure to the CDIM curriculum.

**Methods:**

Internal medicine subinterns used Yammer, a Health Insurance Portability and Accountability Act (HIPAA)–secure enterprise microblogging platform, to post questions, images, and index conversations for searching. The subinterns were asked to submit 4 posts and participate in 4 discussions during their rotation. Faculty reinforced key points, answered questions, and monitored HIPAA compliance.

**Results:**

In total, 56 medical students rotated on an internal medicine subinternship from July 2014 to June 2016. Of them, 84% returned the postrotation survey. Over the first 3 months, 100% of CDIM curriculum topics were covered. Compared with the pilot year, the scale-up year demonstrated a significant increase in the number of students with >10 posts (scale-up year 49% vs pilot year 19%; *P*=.03) and perceived educational experience (58% scale-up year vs 14% pilot year; *P*=.006). Few students (6%) noted privacy concerns, but fewer students in the scale-up year found Yammer to be a safe learning environment.

**Conclusions:**

Supplementing the subinternship clinical experience with an enterprise microblogging platform increased subinternship exposure to required curricular topics and was well received. Future work should address concerns about safe learning environment.

## Introduction

The traditional internal medicine subinternship relies heavily on experiential learning to ensure adequate exposure to various presenting conditions [1]. Unfortunately, student exposure is limited by several factors, including differing patient populations by clinical site, duty hour restrictions, and seasonal variation in presenting illnesses. A review by the Clerkship Directors in Internal Medicine (CDIM) Subinternship Taskforce suggested that this pivotal transition, which bridges the gap between the undergraduate and graduate medical education, needs a formal curricular structure to ensure adequate and balanced exposure to disease processes [2]. Unfortunately, only 31% of internal medicine subinternship clerkship directors admitted to using a formal curriculum [3] despite the existence of standardized teaching tools [2]. In 2002, the CDIM created an Internal Medicine Subinternship Curriculum, whose second version was released on March 2018 [4,5].

Clerkship learning occurs through various means, and our prior work has demonstrated subintern exposure to the CDIM curriculum topics as follows: 19% from direct patient care, 39% from the discussion of a cointern’s patient, 29% from cross-cover patient management, and 17% during conferences and dedicated teaching rounds [6]. Even more alarming, only 14% of students were exposed to ≥15 (of 17) CDIM training problems and >1 out of every 4 subinterns did not have exposure to 35% (6/17) of the training problems during their subinternship month [6].

As of 2016, over 95% of young adults aged 18-24 years had been using social media on a routine basis, with 85% using 6 or more sites regularly [7]. A recent survey on medical residents revealed multiple potential targets of education and further training, such as Web-based privacy, digital professionalism, and protecting patient health information (PHI) while using social media platforms [8]. Enterprise microblogging utilizes a company Web-based platform combining blogging and instant messaging for company workers to post, edit, and sort text and files privately online with specific coworkers in their organization [9,10]. We piloted the use of a Health Insurance Portability and Accountability Act (HIPAA)-compliant enterprise microblogging platform (Yammer) available at the University of Chicago. Muntz et al [11] have successfully implemented Yammer for distance learning during the third-year internal medicine clerkship with high satisfaction and participation rates and low levels of privacy concerns. Although prior work has demonstrated that social media can be used to positively augment a traditional curriculum with moderate to high levels of satisfaction [12-16], no published study to our knowledge has evaluated the use of an enterprise microblogging platform to enhance the clinical experience of subintern medical students. Building on this fact and considering the distributed nature of the subinternship, we hypothesized that employing an enterprise microblogging platform to build a Web-based learning community can augment traditional patient care and ensure a more robust exposure to patient pathology while promoting discussion and learning.

## Methods

### Study Participants

The study was conducted at the University of Chicago Pritzker School of Medicine. We included 5 core faculty and all internal medicine subinterns over 24 consecutive months. The 4-week long subinternships include general internal medicine, cardiology, or medical intensive care unit services. Rotations commenced at either our academic urban tertiary care hospital or a community hospital affiliate (Northshore Hospital) 29 miles away. The students were required to take an overnight call approximately every fourth day and admit up to 3 patients while carrying a total patient census of up to 6.

All fourth-year students participating in an internal medicine subinternship, whether at the University of Chicago or our affiliate, were strongly encouraged to participate for the duration of their subinternship. All students were provided with a consent script indicating that the surveys were anonymous and de-identified as well as the voluntary nature of the study. The experience consisted of several key components: (1) training on the secure social media platform; (2) student-initiated case discussion on the social media platform; and (3) faculty moderating student case discussions to reinforce concepts and identify teaching pearls.

### Social Media Platform

We built a private Yammer discussion group called UC4 for all the internal medicine subinterns. To join UC4, an institution-specific email domain (eg, @uchicago.edu) and invitation are required. Yammer allows students in the private group to begin a conversation or thread around a specific topic, tag those conversations with supplementary files or images, and index the conversations in a searchable manner. Pilot testing of this software has proven the site’s security and invitation-only functionality [10]. All connections to Yammer are secured via secure sockets layer or transport layer security. The project was granted institutional review board exemption based on research conducted in an established or commonly accepted educational setting that involves normal educational practices, such as research on regular and special education instructional strategies. The study was also approved by our HIPAA Compliance Office.

### Student Requirements

Prior to the start of their subinternship, pilot-year students received an email from a faculty member (IA) describing the Yammer study details. During the scale-up year, all students participated in a face-to-face, 45-minute group training session prior to starting the fourth year and again individually prior to starting their subinternship month. All students were educated on how to de-identify all PHI shared in the discussion group and follow universal HIPAA guidelines for PHI protection. Students learned how to access and use Yammer on a desktop device (loading images and files) and how to download and use the mobile app. The students were encouraged to participate throughout their fourth year of medical school.

During their subinternship, the students were asked to submit 4 original cases for discussion and comment on 4 cases submitted by their peers on the platform. The cases could include an actual patient case, teaching pearls learned, or cross-cover events they experienced (Multimedia Appendix 1).

### Faculty Moderation

Faculty facilitators reinforced key educational points made during the discussion, clarified ambiguous areas, and monitored the process for appropriate sharing of PHI.

### Data Collection

We implemented Yammer in 2014-2015 (pilot year) through a smaller pilot starting late summer. In 2015-2016 (scale-up year), we incorporated Yammer into the required Subinternship Preparatory Course and scaled up to include all internal medicine subinterns. The students completed an anonymous, electronic survey (Google Forms for pilot year and E*Value for scale-up year; Multimedia Appendix 2) on the Yammer experience after completing their subinternship. The survey specifically targeted the frequency of posting and ease of use, perceptions of learning, overall satisfaction, and privacy concerns. Study authors (IA, OH, and VMA) analyzed the discussion threads for the frequency of posting and mapped cases to the CDIM Subinternship Curriculum. We compared results from both the pilot and scale-up years to assess implementation success and sustainability. Two-sample tests of proportions were used to compare perceptions between the pilot and scale-up years. Statistical tests were performed using Stata 14.0 software (College Station, TX).

## Results

All 56 fourth-year medical students who rotated on an eligible medicine subinternship during 2014-2016 (n=21 in the pilot year and n=35 in the scale-up year) were included. On average, 41% (23/56) of the subinterns rotated at our affiliate hospital, while 59% (33/56) rotated at the academic hospital; >90% of the students were able to meet the threshold of 4 original posts and commenting on 4 discussions. Within the first 3 months of the pilot year, 100% (17/17) of the CDIM Subinternship Curriculum Topics (“training problems”) had been discussed on the Yammer forum.

**Figure 1:**
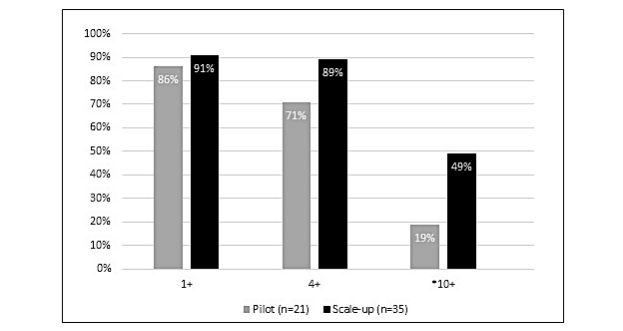
Frequency of posts on Yammer: pilot year versus scale-up year. Asterisk indicates *P*=.03.

Of all the medical students, 84% returned the postsurvey over 2 years (14/21, 67%, in pilot vs 33/35, 94%, in scale-up; *P*=.006). In both years, 87% (41/47) of the students found Yammer easy to use. Usage was high and sustained: 89% (50/56) posted at least once, 82% (46/56) posted 4 or more times, and 38% (21/56) posted ≥10 times. Posting rates were significantly higher in the scale-up year (17/35, 49%) than in the pilot year (4/21, 19%) for ≥10 posts (*P*=.03; Figure 1).

**Figure 2:**
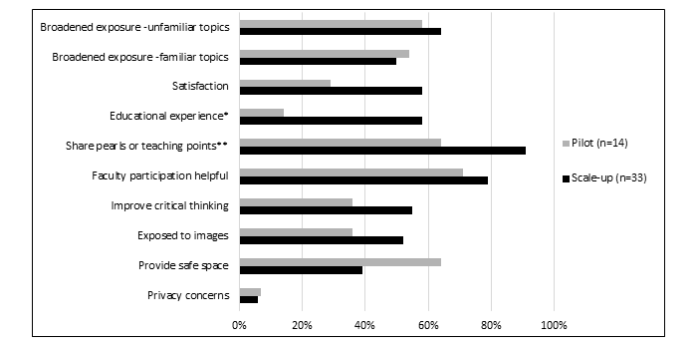
Student perceptions of Yammer: pilot year versus scale-up year. Asterisk indicates *P*=.006; double asterisk indicates *P*=.02.

At least half of the students in both years agreed that Yammer broadened their exposure to both familiar (7/14, 50%, pilot vs 18/33, 55%, scale-up) and unfamiliar (9/14, 64%, pilot vs 20/33, 58%, scale-up) topics (Figure 2). Compared with the pilot year, significantly more students in the scale-up year reported that it was a useful way to share pearls and teaching points (9/14, 64%, pilot vs 30/33, 91%, scale-up; *P*=.02; Figure 2) and rated the educational experience as higher (2/14, 14%, pilot vs 19/33, 58%, scale-up; *P*=.006; Figure 2). More students were satisfied in the scale-up year than in the pilot year (4/14, 29%, pilot vs 19/33, 58%, scale-up; *P*=.07; Figure 2), although the numbers did not reach statistical significance.

A minority of students expressed privacy concerns (1/14, 7%, pilot vs 2/33, 6%, scale-up). Although the numbers were not statistically significant, fewer students felt that Yammer provided a “safe space,” or safe learning environment, in the scale-up year (13/33, 39%) than in the pilot year (9/14, 64%; *P*=.12).

Constructed response feedback from the students and faculty highlighted additional issues for exploration and feedback. The students commented the following: “liked the little pearls and discussing cases,” “many students will really enjoy this forum,” “more enjoyable part of my Sub-I,” “not enough participants,” “forced effort,” and “burdensome.” Faculty commented the following: “I was impressed with the effort the students put into the cases as well as how engaged the responses were. I particularly enjoyed the quality or safety topics,” “many cases generated fruitful discussions, which spurred self-directed and case-directed learning by students,” and “the students were fast to post the most up-to-date clinical trials or guidelines and initiated thoughtful discussions of the findings and relevance to personalized patient care.”

## Discussion

### Principal Results

To the best of our knowledge, this is the first study to evaluate the implementation of an enterprise microblogging platform for the subinternship across geographically disparate sites. Compared with the pilot year, the scale-up year showed an increased frequency of posting and improved student perceptions of educational value. All 17 of the CDIM Subinternship Curriculum topics were discussed within the first 3 months of the pilot year, far higher than our prior experience with personal patient care, cross-cover patient care and conference exposures [6]. Despite these positive outcomes, there are some concerns surrounding the platform’s ability to provide a safe learning environment among colleagues.

Our findings highlight the importance of sustaining new technologies in medical education to provide benefits to learners. There are several reasons why the scale-up year may have been better received than the pilot year. First, a known “cost of building it” exists as the site did not have any background content during its pilot year, whereas the scale-up year had the entire catalog of the pilot-year postings (including images, documents, and links) for students to review. Second, the scale-up year benefited from a timely in-person Yammer orientation during our annual Subinternship Preparatory Course in June, 1 month prior to the start of the first subinternships, as well as a once-a-month refresher orientation prior to the start of each monthly rotation. Last, during the scale-up year orientations, faculty (IA) noted constructive feedback from pilot-year students, such as frustration with long-winded cases without resolution; preference for brief, high-yield pearls and images; and benefits of robust participation with hopes of embedding these lessons into the scale-up year experience.

Use of such technologies involves a certain degree of skepticism. For example, not all students considered Yammer a safe learning environment even though it was a private group. There may be a fear of judgement on their perceived knowledge or skills based on their post content or lack of clarity on who has access to the site. Future work should explore how to better address student concerns to overcome this challenge, particularly for private platforms. Anonymous posting is one option, although that may affect conversation tracking and monitoring of individual student participation. Using student peer social media champions as platform facilitators is another possibility.

### Limitations and Future Directions

Our study was conducted using only one mobile technology platform (Yammer), with students from one medical school rotating in one specialty, the internal medicine subinternship. These limitations may affect the generalizability of our findings. The survey response rate in the pilot year was lower than optimal. Other factors affecting participation include prior use of and comfort with social media, competing responsibilities during the internal medicine subinternship, overall low numbers of subinterns in any given month (maximum 6), and the asynchronous nature of the platform.

Using Kirkpatrick’s training evaluation model (reaction, learning, behavior, and results), we assessed students’ reactions, but future work should address the three other factors [17]. We hope that students are learning with Yammer, but this may require building direct e-learning of Alliance for Academic Internal Medicine (AAIM) Subintern Curriculum 2.0 content and assessing uptake and retention through internal testing or tracking USMLE (United States Medical Licensing Examination) Step 2 scores. Yammer monitors individual student use, but any long-term change to social media use (behavior) would likely have to be assessed through self-reporting. Finally, the ultimate result would be improved quality of patient care; this could be assessed through simulation or directly observed patient care.

We propose the following four strategies to scale up mobile technology and distance learning: (1) engaging a student champion, or peer educator, with social media savvy to improve student satisfaction and participation; (2) adding incentives, possibly gift cards, as posting rates and peer-nominated “best posts” have been shown to improve learner participation [18]; (3) expanding the study to all fourth-year or even all third-year medical students (eg, we have partnered with our third-year medical school internal medicine clerkship directors to utilize Yammer to create a patient advocacy blog and plan to work with our colleagues in the infectious disease field to create a unique mobile learning experience surrounding antibiotic stewardship); and (4) making the initiative multi-institutional. We have partnered with three academic centers to create an e-learning internal medicine subinternship curriculum geared toward the CDIM training cases. This curriculum is hosted on a Web-based platform for distance learning and collaboration. Our goal is to broaden exposure to the Association of American Medical Colleges Core Entrustable Professional Activities and ensure early exposure to American Council for Graduate Medical Education Competencies, a priority of the AAIM CDIM Subinternship Task Force and Association of Program Directors in Internal Medicine collaboration [19,20].

### Conclusions

Supplementing the required clinical experiences in an internal medicine subinternship with an enterprise microblogging platform was feasible, and the majority of students found it easy to use. Overall, Yammer was a well-received addition to the traditional subinternship experience and facilitated distance learning across multiple clinical sites.

## Appendix

Multimedia Appendix 1 Sample screenshots of the interactive microblogging platform.

Multimedia Appendix 2 Survey instrument.

## Abbreviations

AAIM: Alliance for Academic Internal Medicine
CDIM: Clerkship Directors in Internal Medicine
HIPAA: Health Insurance Portability and Accountability Act
PHI: patient health information
USMLE: United States Medical Licensing Examination
