# Supplementary material for: Enterprise Microblogging to Augment the Subinternship Clinical Learning Experience: A Proof-of-Concept Quality Improvement Study
Source: JMIR Med Educ. 2018 Aug 21;4(2):e18. doi: 10.2196/mededu.9810 (PMC6123538; doi:10.2196/mededu.9810)
Supplement: Multimedia Appendix 1 [file mededu_v4i2e18_app1.pdf]

Hey Gang, here is a clinical situation and I'm interested in knowing what you all think are the next moves...

65Y male with a PMH of HTN presenting with SOB. Pt reports that his SOB began 1 week ago. He said that it started off with DOE and a cough which led him to believe he had the flu. He reports coming the ED because he wasn't able to sleep. He reports +orthopnea, +PND, +green/yellow productive cough, +increased b/l LE edema. He denies any changes in diet. Pt reports at baseline he able to walk multiple blocks (approx 2 weeks ago) but would only be able to walk 1 now without stopping. He denies taking any medications and stopped taking his Vasotec after his PCP said the he could control his BP with diet. He denies any CP, chills, night sweats, n/v or recent sick contacts. Denies any drug/alcohol abuse. His bedside CXRs are attached. What labs do we want to order? What is our Ddx? ‹ collapse

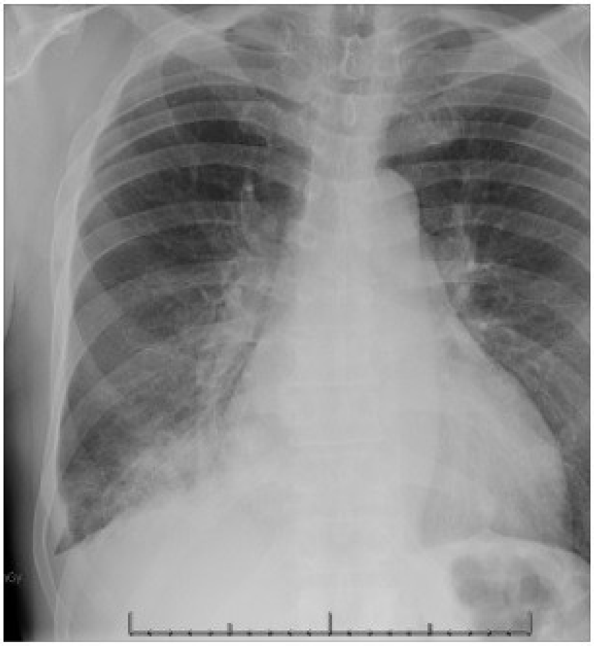

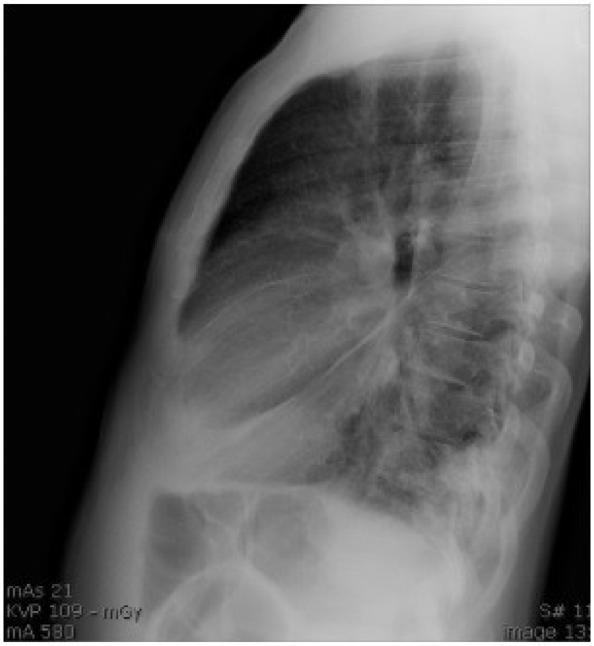

LIKE REPLY SHARE ···

Can I cheat and get the vitals and physical exam? Sounds like a case of ADHF. Does the CXR show some airway filling in the inferior aspect of the right middle lobe?

LIKE REPLY SHARE ···

[redacted] – August 26, 2016 at 12:24am

He's Afebrile, R-24, BP 150/110, HR-97, Physical EXam: only pertinent positives are Cardiac: RRR with nl s1, s2 w/s3, no murmur, no rub, +2 b/l pitting LE edema, JVP elevated to below the ear, +2 distal pulses; Resp: b/l crackles in bases, decreased air exchange, increased effort.

SaO2? Agree, with BP and exam concerning for CHF. The productive cough and R>L lower lobe infiltrate(s) (and associated R diaphgram/R heart border hazziness) could from infectious etiology but no f/c/NS. Could not appreciate lg effusion on cxr. Maybe some pulm vasc congestion and vessel cephalization. Fluid in fissures on lat view. Attaching old, cool study assessing dyspnea in ED pts. Table 3 shows useful +LR and -LR. Recall +LR 10 or higher or -LR 0.1 or lower best!

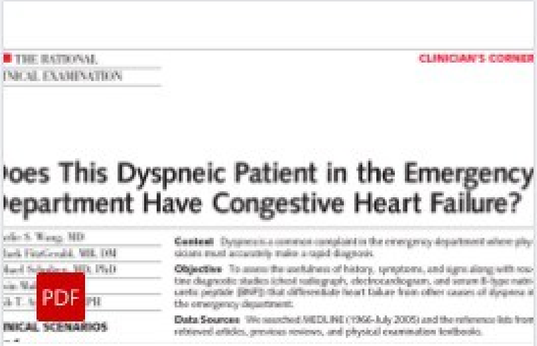
THE RATIONAL
[illegible]NICAL EXAMINATION

CLINICIAN'S CORNER

[illegible]oes This Dyspneic Patient in the Emergency [illegible]epartment Have Congestive Heart Failure?

[illegible]

[illegible]NICAL SCENARIOS

Context Dyspnea is a common complaint in the emergency department where phy[illegible] sicians must accurately make a rapid diagnosis.

Objective To assess the usefulness of history, symptoms, and signs along with rou[illegible] tine diagnostic studies (chest radiograph, electrocardiogram, and serum B-type natri[illegible] uretic peptide [BNP]) that differentiate heart failure from other causes of dyspnea i[illegible] the emergency department.

Data Sources We searched MEDLINE (1966-July 2005) and the reference lists from retrieved articles, previous reviews, and physical examination textbooks.

PDF

Is this CHF

Forgot to say what I would do! EKG, cardiac enzymes, BNP & TTE etc to assess for HFrEF/HFpEF and etiology. CBC (and other basic labs). Consider blood/sputum cx. Was curious about procalcitonin. I know Mercy ED was using this extensively couple years ago. Never see it ordered here (not sure if can?). Was doing some lit search. Interesting article published this year in Am J Med (attached). See supp table showing alorithm using BNP and PCT together! PCT 0.1 or higher gives 22%-82% liklihood of PNA (even 22% likely high enough to Rx). Scary zone is "gray BNP" with PCT <0.1 (still 12% poss PNA!). Interesting that 6.6% pts had CHF AND PNA. Carries double the 60/90d mortality vs CHF alone!

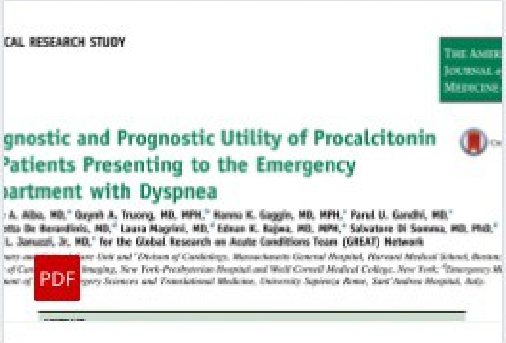
CAL RESEARCH STUDY

gnostic and Prognostic Utility of Procalcitonin
Patients Presenting to the Emergency
partment with Dyspnea

[illegible]

PDF

Hey gang! So you all are correct he does have Heart Failure but he also has CAP. We did a FUS of the heart and could obviously see that he had a decreased EF. So we started him on a HFrEF regimen. We did get a TTE to confirm and just to see what his EF was. We started him on this systolic HF regimen (preload: Lasix, afterload: captopril, carvedilol as his Beta Blocker); this is newly dx so we wanted to also check thyroid function and HIV status; due to CXR showing a Right Lower lobe consolidation, leukocytosis, and purulent sputum we treated for CAP (azithro 500/Ceftriaxone 1g); we also got a RVP, legionella, strep pneumo); we got a resp culture and blood cultures as well.
