# Supplementary material for: Enterprise Microblogging to Augment the Subinternship Clinical Learning Experience: A Proof-of-Concept Quality Improvement Study
Source: JMIR Med Educ. 2018 Aug 21;4(2):e18. doi: 10.2196/mededu.9810 (PMC6123538; doi:10.2196/mededu.9810)
Supplement: Multimedia Appendix 2 [file mededu_v4i2e18_app2.pdf]

THIS FORM IS A PREVIEW ONLY!

There are default headers or footers attached to form types or activities for your program. Please select the activity and form you wish to preview for an accurate view. Please note, the default headers and footers will override any custom text entered in those fields.

{Select form type} {Select activity}

Reload Preview

(Question 1 of 39)

How would you rate your overall satisfaction with the Yammer social media tool as an adjunct to your clinical experience?

- Very dissatisfied
- Neither satisfied nor dissatisfied
- Somewhat satisfied
- Very satisfied

(Question 2 of 39)

How would you rate the educational value of Yammer?

- Very dissatisfied
- Neither satisfied nor dissatisfied
- Somewhat satisfied
- Very satisfied

(Question 3 of 39)

Question 2 continued...

Other:

(Question 4 of 39)

How would you rate your overall satisfaction with the Yammer social media tool as an adjunct to your clinical experience?

- Very dissatisfied
- Somewhat dissatisfied
- Neither satisfied nor dissatisfied
- Somewhat satisfied
- Very satisfied

(Question 5 of 39)

Did Yammer influence clinical decision making or have a direct effect on patient care?

No Somewhat Yes

(Question 6 of 39)

Yammer promoted learning by....

Check all that apply

Selection	Option
☐	Increasing access to attendings to answer clinic questions
☐	Increasing access to peers to answer clinical questions
☐	Allowing students to share "pearls"/teaching points
☐	Exposing me to images (xrays, EKGs etc) I might not have otherwise
☐	Challenged me to think critically when responding to posts
☐	Creating a safe space for discussion of clinical questions
☐	Exposing me to medical literature that was immediately relevant to medical care

(Question 7 of 39)

Yammer broadened my exposure to clinical topics that I had experience with.

- Strongly disagree
- Disagree
- Neither agree/disagree
- Agree
- Strongly agree

(Question 8 of 39)

Question 7 continued...

Other:

(Question 9 of 39)

Yammer broadened my exposure to clinical topics that I did NOT have personal experience with

- Strongly disagree
- Disagree
- Neither agree/disagree
- Agree
- Strongly agree

(Question 10 of 39)

Question 9 continued...

Other:

(Question 11 of 39)

Would you find Yammer to be a useful adjunct on other clinical services?

Definitely not | Probably not | Unsure | Probably | Definitely

(Question 12 of 39)

Question 11 continued...

Other:

(Question 13 of 39)

How easy or hard was Yammer to use?

- Very hard
- Somewhat hard
- Neither easy/hard
- Somewhat easy
- Very easy

(Question 14 of 39)

Question 13 continued...

Other:

(Question 15 of 39)

How much of a burden was Yammer during the rotation?

Very burdensome Somewhat burdensome Not burdensome

(Question 16 of 39)

Question 15 continued...

Other:

(Question 17 of 39)

Faculty participation was prompt, appropriate and helpful

- Strongly disagree
- Disagree
- Neither agree/disagree
- Agree
- Strongly agree

(Question 18 of 39)

Question 17 continued...

Other:

(Question 19 of 39)

I had significant privacy concerns while using Yammer

- Strongly disagree
- Disagree
- Neither agree/disagree
- Agree
- Strongly agree

(Question 20 of 39)

Question 19 continued...

Other:

(Question 21 of 39)

How many "posts" did you submit during your one month subinternship?

- < 5
- 5 - 10
- > 10

(Question 22 of 39)

How many "posts"" did you submit while participating on non-Sub I rotations?

- < 5
- 5 - 10
- > 10

(Question 23 of 39)

Describe your prior use of social media

- Never
- Rarely (once per month)
- Occasionally (once per week)
- Frequently (few times per week)
- Very frequently (daily)

(Question 24 of 39)

Question 23 continued...

Other:

(Question 25 of 39)

Which examples of social media had you used PRIOR to Yammer (check all that apply)?

Selection	Option
☐	Facebook
☐	Twitter
☐	Tumblr
☐	Instagram
☐	Google+
☐	LinkedIn
☐	Doximity

(Question 26 of 39)

Question 25 continued...

Other:

(Question 27 of 39)

Which device did you use to access Yammer (check all that apply)?

Selection	Option
☐	Smartphone
☐	Tablet
☐	Desktop computer
☐	Laptop computer

(Question 28 of 39)

Question 27 continued...

Other:

(Question 29 of 39)

Which operating system(s) did you use to access Yammer (check all that apply)?

Selection	Option
☐	IOS 7
☐	Android
☐	Windows

(Question 30 of 39)

Question 29 continued...

Other:

(Question 31 of 39)

Where did you access Yammer (check all that apply)?

Selection	Option
☐	Home
☐	Hospital
☐	Library

(Question 32 of 39)

Question 31 continued...

Other:

(Question 33 of 39)

Which sub-I service were you on?

- ○ UCMC General Medicine
- ○ UCMC Cardiology
- ○ UCMC Gen Med Transition Team
- ○ Northshore Hospital

(Question 34 of 39)

During which block did you do your subinternship?

- ○ July - September
- ○ October - December
- ○ January - March
- ○ April - June

(Question 35 of 39)

How much time did you spend on Yammer per week?

○ < 10 minutes ○ 10 - 30 minutes ○ 30 - 60 minutes ○ > 60 minutes

(Question 36 of 39)

When did you access Yammer (check all that apply)?

Selection	Option
☐	early morning
☐	mid-day
☐	evening
☐	post call
☐	on call

(Question 37 of 39)

Question 36 continued...

Other:

(Question 38 of 39)

Please describe the strengths and weaknesses of your experience with Yammer

(Question 39 of 39)

Other comments?

Review your answers in this evaluation. If you are satisfied with the evaluation, click the **SUBMIT** button below. Once submitted, evaluations are no longer available for you to make further changes.

Save For Later   Submit
